# Study of wide bandgap SnO_x_ thin films grown by a reactive magnetron sputtering via a two-step method

**DOI:** 10.1038/s41598-022-19270-w

**Published:** 2022-09-12

**Authors:** Y. Zakaria, B. Aïssa, T. Fix, S. Ahzi, A. Samara, S. Mansour, A. Slaoui

**Affiliations:** 1 Qatar Environment and Energy Research Institute (QEERI), Hamad Bin Khalifa University (HBKU), Qatar Foundation, P.O. Box 34110, Doha, Qatar; 2 Laboratoire ICube - CNRS, Université de Strasbourg, 67000 Strasbourg, France

**Keywords:** Materials science, Materials for energy and catalysis, Solar cells

## Abstract

In the present work, we report on the microstructural and optoelectronic properties of SnO_x_ thin films deposited by a reactive radio frequency magnetron sputtering. After SnO_x_ growth by sputtering under O_2_/Ar flow, we have used three different treatment methods, namely (1) as deposited films under O_2_/Ar, (2) vacuum annealed films ex-situ, and (3) air annealed films ex-situ. Effects of the O_2_/Ar ratios and the growth temperature were investigated for each treatment method. We have thoroughly investigated the structural, optical, electrical and morphology of the different films by several advanced techniques. The best compromise between electrical conductivity and optical transmission for the use of these SnO_x_ films as an n-type TCO was the conditions O_2_/Ar = 1.5% during the growth process, at 250 °C, followed by a vacuum post thermal annealing performed at 5 × 10^–4^ Torr. Our results pointed out clear correlations between the growth conditions, the microstructural and optoelectronic properties, where highly electrically conductive films were found to be associated to larger grains size microstructure. Effects of O_2_/Ar flow and the thermal annealing process were also analysed and discussed thoroughly.

## Introduction

Transparent conductive oxides (TCOs) are imperative materials in various technologies requiring optical transparency and electrical conductivity. In addition to these two characteristics, TCOs combine a third property, namely a high chemical stability. More specifically, owing to its optoelectronic properties and its production scalability, tin-doped indium oxide (ITO) is a preferred TCO for industrial applications and it has been extensively employed as transparent electrodes for various solar cells technologies, light emitting diodes and flat panel displays^1–11^. However, researchers are working on the development of alternatives materials to ITO due to the Indium (In) supply challenges in the future. Tin (Sn), instead, is a low-cost material of much higher earth abundance than Indium (In). Two stoichiometric tin oxide compounds, namely SnO and SnO_2_, are known to be wide band gap oxide semiconductors with tetragonal litharge and rutile type structures, respectively. Tin in SnO_x_ has two chemical states of Sn^2+^ for SnO phase and Sn^4+^ for the SnO_2_ one. The first is intrinsically p-type semiconductor while the second is n-type^12,13^.

In particular, SnO_x_ has been largely investigated in applications of gas sensors, solar cells, transparent electrodes, and thin film transistors^11,14–20^. Moreover, in the past decades, SnO was the key material for anode materials^21^, coatings^22^, catalysis^23^, and precursors for the production of SnO_2_^24,25^, because of its properties of gas-sensitivity and metastability to transform into SnO_2_ at O_2_-rich ambient. Recently, SnO has been drawn back into attention mainly because of the difficulty in obtaining high-quality p-type such as doped ZnO^26,27^, NiO^28,29^, Cu_2_O^30^. Previous studies show that the maximum hole mobility of SnO films is about 2.6 cm^2^/V s, fairly high among p-type conductive oxides, and it can be further improved via proper doping^19^. Those properties render SnO a promising candidate to be a next p-type oxide semiconductor for advanced optoelectronic devices. Several techniques have been used to grow SnO_x_ films on various substrates, including reactive RF magnetron sputtering^31^, e-beam evaporation^18^, laser ablation^25^, and atomic layer deposition^32^. However, the prepared SnO_x_ films are often mixed with some impurity phases, including metallic Sn, and intermediate oxides containing both 2^+^ and 4^+^ valences^31,33,34^. The reason is that SnO can decompose according to the disproportion at ion reaction even in the absence of oxygen at suitable temperature^16,17^. Consequently, deposition conditions and growth temperature are of great importance in the fabrication of single phase SnO_x_ films.

In the present work, polycrystalline SnO_x_ thin films were fabricated on quartz substrates by a two-step method, i.e., RF MS from high-purity SnO source target first and subsequent air and vacuum annealing treatments. The morphology, crystal phase, chemical composition, optical, and electrical properties of the obtained SnO_x_ thin films were characterized by Grazing Incidence X-ray Diffraction (GIXRD), Scanning Electron Microscopy (SEM), Transmitting Electron Microscopy (TEM), UV–visible, and Hall effect measurement, as detailed in the experimental section. The depth profiling of the various elements was also investigated by time of flight-second ion mass spectroscopy (TOF-SIMS) throughout the thickness of the films. The results show in detail the effect of the oxygen flow and the temperature during the growth on the microstructure and eventually on the electrical conductivity and the optical transmittance. The optimized conditions were clearly defined and discussed thoroughly. The majority of the samples exhibit an average optical transmittance with more than 80% between 400 and 700 nm, while the highest conductive thin films are dense, with large grain size and without pinholes and/or cracks.

## Experimental section

### Materials and methods

The SnO_x_ thin films were prepared in two subsequent steps. The first step consists of the RF magnetron sputtering (manufactured by Torr) of a high purity SnO (99.99%) 2″- diameter target (Manufactured by Codex International) on 1″ × 3″ cleaned quartz substrates at two different temperatures, namely 100 and 250 °C, under different O_2_ to Ar flow rates ratios, namely O_2_/Ar = 0, 0.5, 1.5, 2.5, 4.5 and 7.5%. Each sample was cut into three 1″ × 1″ smaller samples. The second step consists of two different post deposition annealing at 400 °C for 1 h, one in air and the other one in a controlled moderate vacuum (with a constant Ar flow of 5 sccm) at 5 × 10^–4^ Torr.

The depositions conditions using RF magnetron sputtering were: 50 W power, 200 sccm of Ar flow and 30 min deposition time for all samples. The first batch was deposited at 100 °C, and the second batch was deposited at 250 °C. Prior the deposition, the base pressure of 5 × 10^–5^ Torr was first achieved, and the deposition pressure, which depends on the variable oxygen flow rate, varied from 4.4 × 10^–3^ to 5.1 × 10^–3^ Torr.

### Materials characterizations

Several characterization techniques were employed to investigate the micro-structure, crystalline structure, optical transmittance and bandgap, resistivity, charge carrier concentration and mobility, and elemental depth profiles.

GIXRD has been conducted using Rigaku - Smartlab. The x-ray source Cu K-alpha at 1.54 Å, the 2θ scans of the detector are from 15 to 65°, the step and the speed are 0.02° and 2°/min while the x-ray incident beam was kept at 0.55°. UV–Visible Spectroscopy was performed using Perkin Elmer - Lamda 1050, using 4 nm steps. Electrical properties were conducted for all samples using benchtop four-point probe system and Hall effect - Lakeshore 8400. Depth profiles were obtained using ToF-SIMS-IONTOF. Scanning/Transmission Electron Microscopy (SEM/TEM) images were obtained using FEI - Quanta 650/Talos, respectively. TEM lamella has been prepared by Focused Ion Beam (FIB)/SEM. The process consists of coating the SnO_x_ film by a protective Pt layer. Then the whole stack Pt/SnO_2_/Substrate undergoes the thinning process to reach a convenient vertical thickness for the TEM imaging. TOF-SIMS analysis was performed by positive Bi^+^ ion primary beam at 30 keV and ~ 1.3 pA current over a 100 × 100 μm^2^ analysed area using random rastering mode while the sputtering was achieved using Cs^+^ ion beam at 2 keV over 400 × 400 μm^2^. The depth profile has been conducted in positive polarity which targets the positive ions emerging from the surface at each etched level.

## Results and discussions

Following a first visual observations just after the SnO_x_ deposition, all the samples looked homogenous and most of the films had the yellowish colour which is a typical characteristic of SnO_x_ material.

### GIXRD analysis of as-deposited samples

**Figure 1:**
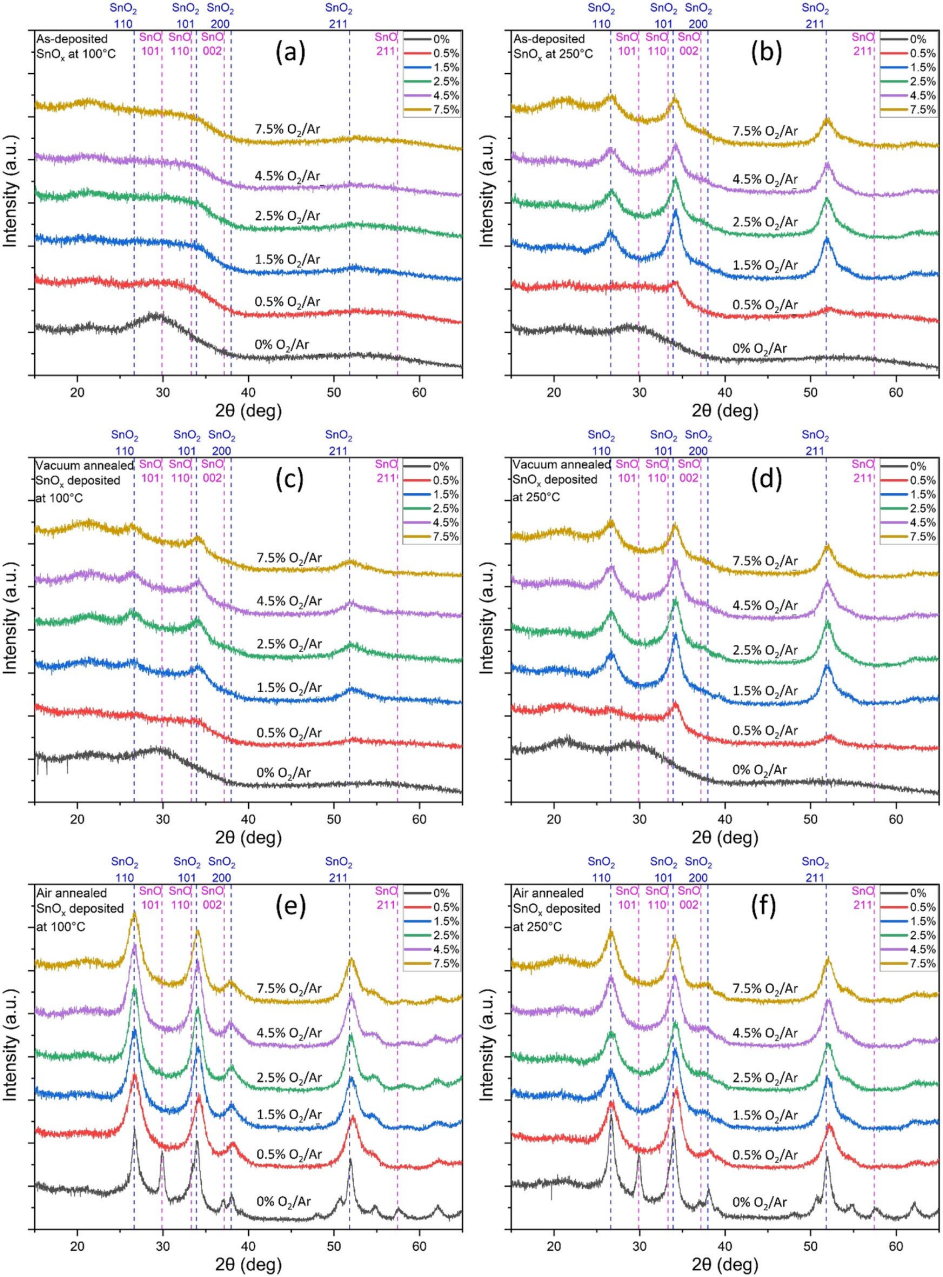
GIXRD patterns of SnO_x_ samples (**a**) as deposited (as-dep) at 100 °C, (**b**) as-dep at 250 °C, (**c**) as-dep at 100 °C and annealed in vacuum at 400 °C, (**d**) as-dep at 250 °C and annealed in vacuum at 400 °C, (**e**) as-dep at 100 °C and annealed in air at 400 °C, (**f**) as-dep at 250 °C and annealed in air at 400 °C.

For the as-deposited SnO_x_ at 100 °C, the samples do not show any XRD sharp peak, thereby indicating the low crystalline quality and/or the amorphous microstructure of this series (Fig. 1a). The sample deposited without O_2_ flow has a broad peak at around 30° revealing the presence of a very low crystalline microstructure of SnO. The XRD pattern for SnO_x_ samples deposited in presence of O_2_ show a low crystalline SnO_2_ phase due to the shift of the broad peak from 30 to 34°. It is worth to note that the XRD pattern did not change while increasing the O_2_/Ar ratio from 0.5% to 7.5%, which may indicate that increasing the O_2_ flow does not improve the crystalline microstructure of SnO_x_ at this deposition temperature of 100 °C.

For as-deposited SnO_x_ at 250 °C (Fig. 1b), the samples deposited at O_2_/Ar ratios equal or above 0.5% have a XRD pattern which shows at least one peak, and the most intense peak located at 34° and corresponding to the (101) plane is observed for deposition at O_2_/Ar = 1.5%. This sample deposited at O_2_/Ar = 1.5% also exhibits the highest crystallite size corresponding to the (110) plane is 56 Å. The crystallite size decreases to 45 nm while increasing the O_2_/Ar from 1.5 to 4.5%. The crystallite size was not calculated for the other samples due to peak definition as shown in Table S2 (supplementary information). It is also expected that the growth conditions with the absence of O_2_ flow would favour the presence of SnO due to the material of the sputtering target. Furthermore, the growth under O_2_ flow has formed a SnO_2_ phase and hence, increasing the growth temperature has clearly improved the crystalline quality of SnO_2_ as concluded from XRD analysis.

### GIXRD analysis of thermally annealed samples

For SnO_x_ samples deposited at 100 °C and annealed at 400 °C under moderate vacuum, similarly to as-deposited samples at 100 °C without O_2_ flow, XRD pattern shows a broad peak at around 30° revealing the presence of low crystalline SnO for the sample deposited without O_2_ flow (Fig. 1c). The XRD pattern also shows a broad peak at around 34° related to the presence a low crystalline SnO_2_ phase for the samples deposited in the presence of O_2_. For O_2_/Ar above 0.5%, there is a small peak located at 34° and associated to the (101) plane. No noticeable change was then observed while varying the O_2_/Ar.

For SnO_x_ samples deposited at 250 °C and annealed at 400 °C under moderate vacuum, similarly to previous samples deposited without O_2_ flow, there is a clear indication of a low crystalline SnO phase (Fig. 1d). While varying the O_2_/Ar from 0.5 to 7.5%, there is a clear crystalline microstructure as indicated by XRD pattern. The crystallite size related to SnO_2_ (110) peak decreases from 61 to 56 Å when O_2_/Ar goes from 1.5 to 2.5% and then it remains constant for higher O_2_/Ar ratios as revealed by Table S1 (supplementary information). It was clear that the vacuum annealing has improved the crystallinity of the SnO_x_ samples deposited in presence of O_2_.

For SnO_x_ samples deposited at 100 °C and annealed at 400 °C under air, all samples show a remarkable improved crystallinity (Fig. 1e). For air annealed SnO_x_ deposited without O_2_ flow, there are two high crystalline phases of SnO and SnO_2_ as revealed by the presence of sharp peaks located at 29.9° indicating SnO and at 33.9° indicating SnO_2_. The crystallite size related to SnO_2_ (110) peak drastically decreases from 116 Å to 51 Å when O_2_/Ar goes from 0 to 0.5%. Then, it increases slightly up to 62 Å when O_2_/Ar varies from 0.5 to 2.5% and it decreases to 57 Å when O_2_/Ar goes from 2.5 to 7.5%. The sharp peak located at 26.6° and corresponding to the (110) plane of SnO_2_ deposited without O_2_, represents the highest crystallite size of all the deposited SnO_x_ samples.

For SnO_x_ samples deposited at 250 °C and annealed at 400 °C under air, all samples show an improved crystallinity compared to as-deposited SnO_x_ (Fig. 1f). For air annealed SnO_x_ deposited without O_2_ flow, there are also two high crystalline phases of SnO and SnO_2_ as revealed by the presence of sharp peaks located at 29.9° indicating SnO and at 33.9° indicating SnO_2_. The crystallite size of SnO_2_ drastically decreases from 109 Å to 54 Å when O_2_/Ar goes from 0 to 0.5%. Then, it slightly stabilizes around the value of 53 Å when O_2_/Ar goes from 0.5 to 7.5%. Air annealing has clearly improved the crystallinity of SnO_x_ samples which were deposited at 100 °C compared to vacuum annealing. It is worth to note that SnO peaks were only observed in both air annealed samples deposited with O_2_ flow.

The as-deposited samples have shown that the relatively high temperature of 250 °C (i.e. as compared to 100 °C) has improved the crystalline microstructure for all the samples deposited with the presence of O_2_. This is due to the improved crystallization of SnO_x_ and the reduction of the microstructure disorder^31^. Vacuum annealing has then slightly improved the crystallinity for samples deposited at 100 °C due to the higher annealing temperature of 400 °C. However, there is no clear improvement of the crystalline microstructure of the samples deposited at 250 °C. The air annealing has improved remarkably the SnO_x_ microstructure for all samples deposited at 100 °C due to the higher annealing temperature of 400 °C, compared to the deposition temperature and to the abundant presence of O_2_ which enabled the crystallization of SnO_x_. Air annealing for SnO_x_ sample deposited at 250 °C without O_2_ has improved substantially the crystallinity of SnO_x_ due to the presence of O_2_ at higher temperature of 400 °C. Moreover, further crystallization was less extensive under air annealing for other samples deposited in presence of O_2_. This is likely due to the small temperature gradient between the deposition and the annealing processes, as well as the presence of O_2_ in both processes. SnO crystallinity was not achieved only by depositing SnO without O_2_ at both temperatures of 100 and 250 °C, as well as after annealing these samples under vacuum. However, SnO phase appeared in both samples deposited at 100 and 250 °C without O_2_ and annealed under air. This indicates that SnO crystallinity may be improved using a controlled annealing process under atmospheric pressure and in absence of O_2_^31,32,35–38^. The different observations extracted from XRD are summarized in Table 1.

**Table 1:** Summary of SnO_x_ crystallinity samples using XRD and Scherrer equation for peak (101).

O_2_/Ar ratio (%)	Deposition temperature (°C)					
	100	250	100	250	100	250
	As deposited		Vacuum Annealing at 400 °C		Air Annealing at 400 °C	
0	(SnO) Low crystallinity	(SnO) Low crystallinity	(SnO) Low crystallinity	(SnO) Low crystallinity	* Higher crystallinity	Higher crystallinity
0.5	Low crystallinity	Poorly crystallised	Poorly crystallised	Lower crystallinity	Lower crystallinity ↓ High crystallinity ↓ Lower crystallinity	Average crystallinity
1.5		Average crystallinity ↓ Lower crystallinity		+ High crystallinity ↓ Lower crystallinity		
2.5						
4.5						
7.5						

+ best conductivity, * best crystallinity.

### Electrical transport properties

The electrical properties were investigated initially using the four-point probe IV tool then through the Hall effect measurements. Four-point probe measurements were performed on all samples using three-point statistics method. The obtained results revealed a very high resistivity around 10^8^ Ω cm for SnO_x_ deposited without the presence of O_2_ for both as-deposited series at 100 °C and 250 °C. As revealed by XRD results, this may be attributed to the low crystallinity of the SnO_x_ films. The resistivity decreases to its lowest value of 47 Ω cm at O_2_/Ar = 0.5% for SnO_x_ samples deposited at 100 °C and to its lowest value of 4.5 Ω cm at O_2_/Ar = 1.5% for SnO_x_ samples deposited at 250 °C. The lower value of resistivity for samples deposited at 250 °C is matching with the highest crystallite size which indicated as discussed earlier the improvement of the crystalline microstructure.

**Figure 2:**
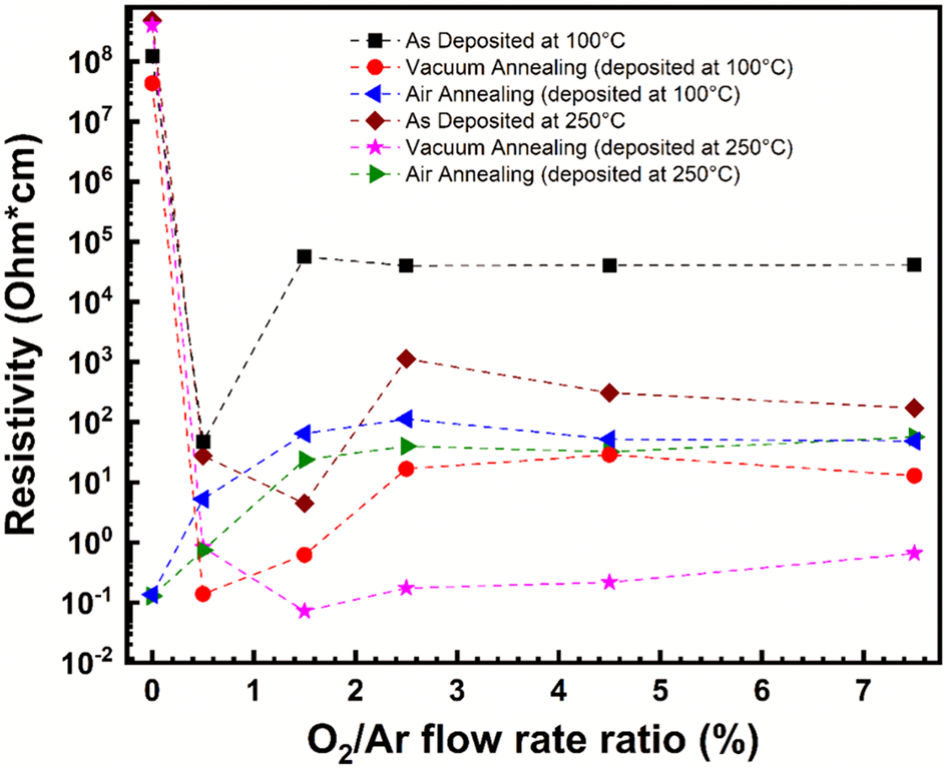
Resistivity as a function of O_2_ flow in growth conditions for as-deposited, vacuum-annealed, and air-annealed samples.

For SnO_x_ as deposited at 100 °C, the resistivity increases significantly from O_2_/Ar = 0.5 to 1.5% and it relatively stabilizes at higher O_2_/Ar starting from 1.5% (Fig. 2). For SnO_x_ as deposited at 250 °C, the same behaviour is observed, where the resistivity increases significantly from O_2_/Ar = 1.5 to 2.5% and it relatively decreases towards higher O_2_/Ar starting from 2.5%. In presence of O_2_, all deposited SnO_x_ samples at 250 °C show a better electrical conductivity compared to films grown at 100 °C.

Vacuum annealed samples were found to follow the similar pattern of as-deposited SnO_x_ at both deposited temperatures where the best conductive as-deposited SnO_x_ samples are still the best conductive ones. Moreover, the recorded resistivity values are 0.14 Ω cm for the best SnO_x_ deposited at 100 °C and 0.07 Ω cm for the best SnO_x_ deposited at 250 °C of all the fabricated batches after vacuum annealing. Furthermore, as expected after annealing process, all the recorded resistivity is significantly lower compared to the as-deposited ones. This improvement is likely attributed to the microstructure improvement and to the conservation of charge carrier after the vacuum annealing of the samples.

For both air annealed series, the lowest resistivity of around 0.13 Ω cm series was observed for samples deposited without O_2_ flow. The resistivity increases then significantly until O_2_/Ar = 1.5% and slightly stabilizes for higher O_2_/Ar starting from 1.5%. The trend of resistivity with respect to O_2_/Ar (of both air-annealed series) are very closely matching regardless the deposition temperature of SnO_x_. However, the resistivity for SnO_x_ samples deposited at 250 °C is slightly lower for all O_2_/Ar ratios except the two extreme values of 0 and 7.5% as shown in Fig. 2.

The samples deposited at 250 °C followed by a moderate vacuum annealing at 400 °C showed the lowest resistivity of 0.07 Ω cm at O_2_/Ar = 1.5%. Furthermore, the other SnO_x_ samples in the same series deposited with O_2_/Ar ratio above 1.5% show substantially a lower resistivity compared to samples from other series with the same conditions of O_2_/Ar. This can be attributed to the relatively higher crystallite size due to the annealing process as well as the expected charge carrier concentrations due to the vacuum annealing^31,37,38^.

Further analysis using Hall effect measurement were conducted solely on the best conductive samples identified by four-point probe method, both for vacuum and air annealed samples series. The electron mobility for the best conductive samples for each series were 1.07 cm^2^/V s for vacuum annealed series deposited at 100 °C, 7.77 cm^2^/V s for vacuum annealed series deposited at 250 °C, 2.11 cm^2^/V s for air annealed series deposited at 100 °C, and 2.58 cm^2^/V s for air annealed series deposited at 250 °C. Their respective charge carrier concentrations were 1.47 × 10^19^ cm^−3^ for vacuum annealed series deposited at 100 °C, 5.84 × 10^18^ cm^−3^ for vacuum annealed series deposited 250 °C, 1.39 × 10^19^ cm^−3^ for air annealed series deposited at 100 °C, and 1.26 × 10^19^ cm^−3^ for air annealed series deposited at 250 °C. All these conductive samples were n-type semiconductors which is expected for vacuum annealed samples due to the presence of SnO_2_ phase. All the results are summarised in Table 2.

**Table 2:** Hall effect measurements for SnO_x_ samples of each annealed series.

Series	Best conductive O_2_:Ar (%)	Mobility (cm^2^/V s)	Charge carrier (cm^−3^)
As deposited at 100 °C/Vacuum annealed	0.5	1.07	1.47 × 10^19^
As deposited at 250 °C/Vacuum annealed	1.5	7.77	5.84 × 10^18^
As deposited at 100 °C/Air annealed	0	2.11	1.39 × 10^19^
As deposited at 250 °C/Air annealed	0	2.58	1.26 × 10^19^

However, the n-type conductivity for air annealed samples reveals that the majority charge carriers are related to the SnO_2_ phase. The most electrically conductive SnO_x_, which is deposited at 250 °C/O_2_/Ar = 1.5% and annealed under vacuum at 400 °C is a result of a much higher mobility and an average charge carrier concentration compared to other samples.

Normally, scattering mechanisms are the main explanation for the electron mobility. Furthermore, as per the grain-boundary scattering mechanism, the mobility increases while increasing the carrier concentration or the crystallite size. However, for higher values than 10^20^ cm^−3^ of charge carrier concentration, the mobility decreases due to the domination of ionised scattering mechanism^36,39,40^. Therefore, the grain-boundary scattering mechanism is likely the main mechanism responsible for the higher electron mobility of the best conductive SnO_x_ sample. As a matter of fact, Kim et al.^41^ established that the grain boundary scattering was the dominant scattering mechanism for SnO_2−*x*_ thin films prepared by magnetron sputtering^41^. In another work performed on polycrystalline GZO, Hall mobility measurements indicated that the mobility of electron which transports across many grains and grain boundaries in conduction path was limited by both scattering effects in ingrains and at grain boundaries^42^. The dominancy of the scattering effects varies with electron concentration. In the case of higher electron concentration above about 10^20^ cm^−3^, the dominant scattering effect for electron mobility (µ_Hall_) has been considered to be ingrain scattering (ionized-impurity scattering)^43,44^. On the other hand, it is well established that optical mobility (µ_opt_) shows the electron mobility in ingrains. Thus, µ_opt_ is limited by the effect of ingrain scattering. In advanced investigations, comparing electron and optical mobilities has been employed to highlight the contribution of grain boundary scattering on electron mobility, and this approach has frequently been used as a means to study the effect of ingrain and grain boundary scattering on electron transport properties in many TCOs^45–50^.

### Optical properties

The optical properties have been studied using UV–Visible spectroscopy. The optical transmittance measurements were conducted on all the grown samples and the average optical transmittance between 400 and 700 nm is summarized in Table 3. The average optical transmittance of the reference Quartz substrate was initially measured at 93%. For SnO_x_ samples deposited at 100 °C, it is observed that the average optical transmittance from 400 to 700 nm (labelled transmittance) increases from 72 up to 89% when O_2_/Ar increases from 0 to 7.5%. Moreover, the transmittance increases from 70 to 89% after vacuum annealing and increases from 65 to 91% when O_2_/Ar varies from 0 to 7.5% after air annealing. For SnO_x_ samples deposited at 250 °C, the transmittance in as-deposited and vacuum annealed samples decreases from around 80% to 76% when O_2_/Ar varies from 0% to 0.5%. Furthermore, the transmittance increases up to 91% while O_2_/Ar increasing from 0.5% to 7.5%. The transmittance increases from 77 to 91% while O_2_/Ar varying from 0 to 7.5% after air annealing. These results are summarised in Table 3.

**Table 3:** Average optical transmittance between 400 and 700 nm for SnO_x_ samples.

O_2_/Ar ratio (%)	Optical transmittance (%)					
	Deposition temperature (°C)					
	100	250	100	250	100	250
	As-deposited		Vacuum annealing at 400 °C		Air annealing at 400 °C	
0	72	80	70	79	65	77
0.5	78	76	80	76	86	87
1.5	87	87	86	87	88	89
2.5	87	88	87	88	89	89
4.5	88	90	87	89	90	90
7.5	89	91	89	91	91	91

It is established that the oxygen flow has a strong effect on the optical properties of SnO_x_^36^. This can be directly observed by the blue shift of the absorption edge as well as the relative increase of transmittance when O_2_/Ar is increasing as shown in Figure S1 (supplementary information).

Figure S2 (supplementary information) show Tauc plots for all samples considering the SnO_x_ thin films as a direct bandgap semiconductor. It can be concluded from Tauc plots that all the SnO_x_ films have a wide optical band gap varying from 3.3 to 4.5 eV. Table 4 summarised the values of the optical band gap for all the samples, which are found to increase and then stabilise when O_2_/Ar is increasing. The low optical bandgap for the SnO_x_ samples deposited without the presence of O_2_ compared to the other samples, is a typical characteristic for SnO and it was already reported in the literature varying from 2.6 to 3.4 eV^36^. These results corroborate well the XRD analysis and are confirming the major presence and the low crystallinity behaviour of the SnO phase for both the as-deposited and vacuum annealed samples. The measured bandgap above 4 eV for the rest of the samples is related to SnO_2_ phase which is very close to the values reported in the literature. The band gap variation is mainly related to the difference of the stoichiometry of the SnO_x_ films. However, the effect of the sample disorder can also decrease the bandgap of the SnO_x_ thin films^35,36,51^.

**Table 4:** Optical bandgap for SnO_x_ samples.

O_2_/Ar ratio (%)	Optical bandgap (eV)					
	SnO_x_ deposition temperature (°C)					
	100	250	100	250	100	250
	As-deposited		Vacuum annealing at 400 °C		Air annealing at 400 °C	
0	3.4	3.7	3.3	3.6	3.3	3.3
0.5	4.5	4.4	4.2	4.4	4.4	4.4
1.5			4.5			
2.5		4.5				
4.5						
7.5						

The band gap of SnO_2_ samples prepared in presence of O_2_ is relatively very high compared to the literature where all samples reached 4.4–4.5 eV except one sample of SnO_2_ deposited at 100 °C/0.5% O_2_:Ar and annealed under vacuum. These band gap values are related to the high oxidation of SnO_2_ due to the oxidized sputtering target and the presence of oxygen during the growth.

The optoelectronic performance for all SnO_x_ samples was evaluated using Haacke figure of merit (FoM) Eq. (1)^52^. The results are shown in Table 5. SnO_x_ samples have a relatively low figure of merit due to the moderate resistivity of the thin films. The highest figure of merit φ of 5.14 × 10^–2^ (10^–3^ Ω^−1^) is related to our best conductive SnO_x_ sample. Furthermore, the SnO_x_ samples deposited at 250 °C where the O_2_/Ar ratio between 1.5 and 4.5% as well as SnO_x_ sample deposited at 100 °C and at 0.5% O_2_/Ar ratio have shown figure of merit above 10^–2^ (10^–3^ Ω^−1^). These results reveal that the vacuum annealing has improved the optoelectronic performance of the SnO_x_ thin films.

1 $$\upphi = {\text{T}}^{{{1}0}} /{\text{R}}_{{\text{s}}}$$

where φ is figure of merit, T is the average optical transmittance from 400 to 700 nm, and Rs is the sheet resistance.

**Table 5:** Figure of merit for all SnO_x_ samples.

O_2_/Ar ratio (%)	Figure of merit (10^–3^ Ohm^−1^)					
	Deposition temperature (°C)					
	100	250	100	250	100	250
	As-deposited		Vacuum annealing at 400 °C		Air annealing at 400 °C	
0	3.06 × 10^–12^	3.21 × 10^–12^	6.52 × 10^–12^	3.38 × 10^–12^	9.91 × 10^–04^	8.17 × 10^–03^
0.5	4.27 × 10^–05^	3.93 × 10^–05^	**1.87 × 10**^**–02**^	1.32 × 10^–03^	1.02 × 10^–03^	5.70 × 10^–03^
1.5	1.12 × 10^–07^	8.31 × 10^–04^	9.13 × 10^–03^	**5.14 × 10**^**–02**^	1.10 × 10^–04^	1.96 × 10^–04^
2.5	1.72 × 10^–07^	4.45 × 10^–06^	4.10 × 10^–04^	**2.90 × 10**^**–02**^	7.66 × 10^–05^	1.42 × 10^–04^
4.5	1.35 × 10^–07^	1.22 × 10^–05^	1.72 × 10^–04^	**1.56 × 10**^**–02**^	1.33 × 10^–04^	1.17 × 10^–04^
7.5	1.15 × 10^–07^	3.70 × 10^–05^	3.70 × 10^–04^	**9.66 × 10**^**–03**^	1.23 × 10^–04^	1.12 × 10^–04^

Significant values are in [bold].

**Figure 3:**
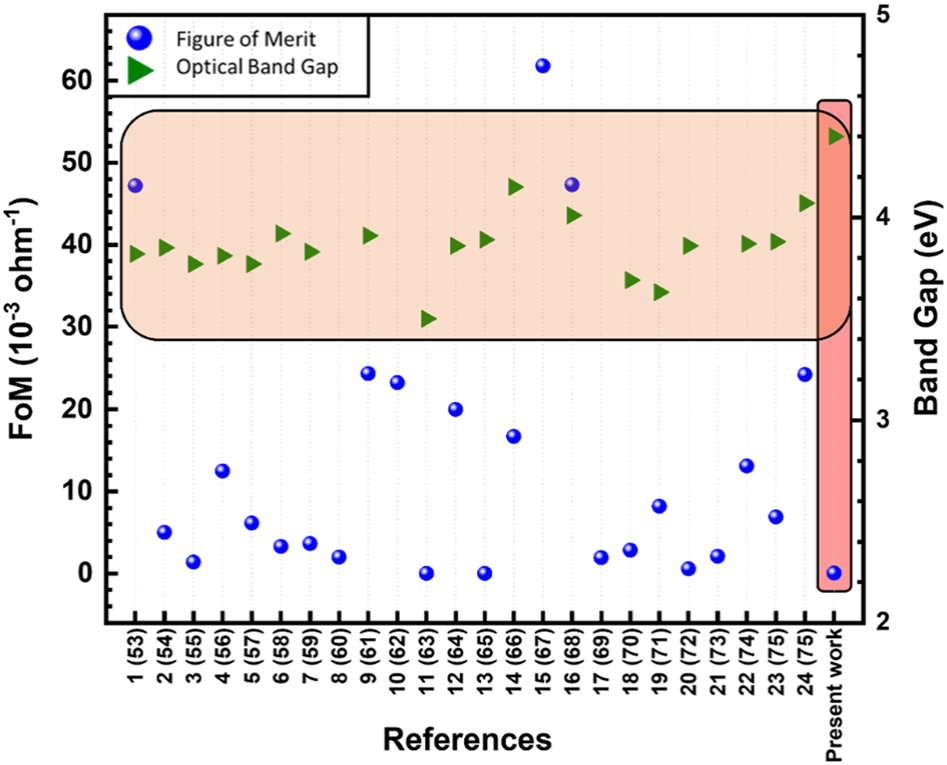
Summary of Figure of Merit values and bang gap as a function of the various references^53–75^.

Table S3 (supplementary information) summarizes selected values from relevant literature of FoM for different doped SnO_2_ thin films along with the doping type, synthesis method, band gap value, electrical resistivity and sheet resistivity, and optical transmittance^53–75^. Only two references of undoped films has been found in addition to our present work. Figure 3 highlights these FoM and band gap values as a function of the various references along with our present work. Highest FoM has been recorded for SnO_2_ grown with spray pyrolysis and doped with fluorine, while the lowest value characterized SnO_2_ deposited by Pulsed Laser Deposition and doped with Tellurium. While our measured FoM belongs rather to the category of low values in Table S3 (supplementary information), which is rather expected since our SnO_2_ films are undoped and did not reach a low resistivity around 10^–4^ Ω cm. However, the optical band gap was among the highest reported in the literature data which is, as discussed previously, due to the high oxidation state of SnO_x_.

### Morphology and structural analyses

**Figure 4:**
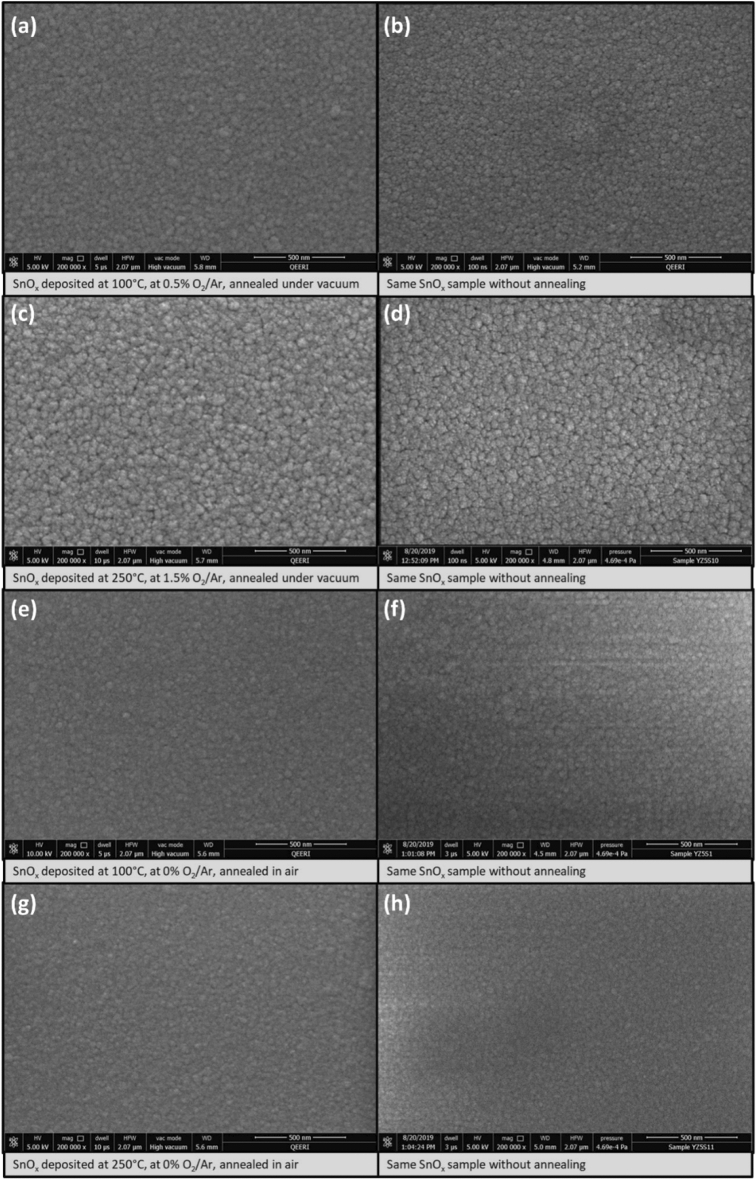
Representative SEM images for the most electrically conductive annealed samples and their related SnO_x_ samples without annealing: (**a**) SnOx deposited at 100 °C, 0.5% O_2_/Ar, annealed under vacuum, (**b**) SnOx deposited at 100 °C, 0.5% O_2_/Ar, without annealing, (**c**) SnOx deposited at 250 °C, 1.5% O_2_/Ar, annealed under vacuum, (**d**) SnOx deposited at 250 °C, 1.5% O_2_/Ar, without annealing, (**e**) SnOx deposited at 100 °C, 0% O_2_/Ar, annealed in air, (**f**) SnOx deposited at 100 °C, 0% O_2_/Ar, without annealing, (**g**) SnOx deposited at 250 °C, 0% O_2_/Ar, annealed in air, (**h**) SnOx deposited at 250 °C, 0% O_2_/Ar, without annealing.

SEM images were conducted on the four samples from the annealed series showing the highest conductivity as well as their related SnO_x_ samples without annealing. All four samples show a crack-free SnO_x_ films. The annealing process did not change drastically the morphology of the SnO_x_ thin films. It can be clearly observed that the best conductive sample deposited at 250 °C with O_2_/Ar = 1.5% and annealed under vacuum (Fig. 4) has the largest grain size compared to the other samples as shown in Fig. 4. The large grain size has improved the conductivity of SnO_x_ as previously reported^36^. Following the discussion regarding the Hall effect results, the high mobility associated to these specific growth conditions is also attributed to the large grain size^33,36^. The sample deposited at 100 °C and with O_2_/Ar = 0.5% and annealed under vacuum (Fig. 4c) has shown the smallest grain size compared to the other three SnO_x_ samples. This result is corroborating well the Hall effect measurement as it has shown the lowest mobility among the four selected samples.

**Figure 5:**
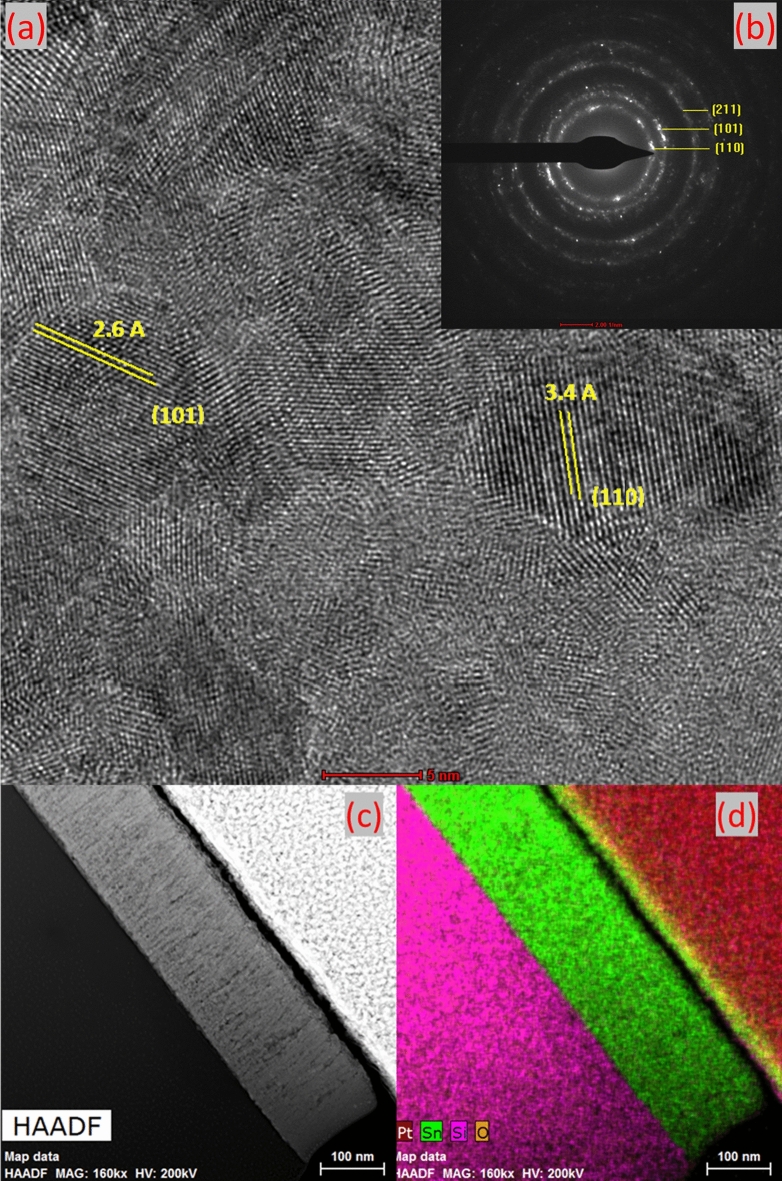
Cross-section TEM images for the best conductive sample (**a**) high resolution TEM image, (**b**) TEM diffraction pattern, (**c**) HAADF image, (**d**) EDS mapping.

In order to confirm the multi-crystalline structure of the best conductive SnO_2_ sample, we performed TEM imaging and mapping (Fig. 5). The interplanar spacing could be measured directly from the image (Fig. 5a) namely (110) and (101) planes, which is matching with the results revealed by XRD. Figure 5b shows the TEM diffraction pattern indexation revealing the intense patterns are related to (110) and (101) planes. High-angle annular dark-field imaging (HAADF) shown in Fig. 5c has revealed a dense SnO_2_ film with elongated column-shape crystalline structure towards the growth direction. EDS mapping (Fig. 5d) has revealed the presence of a uniform layer of SnO_2_ which is forming a sharp and clear interfaces with the quartz substrate^33,36^.

**Figure 6:**
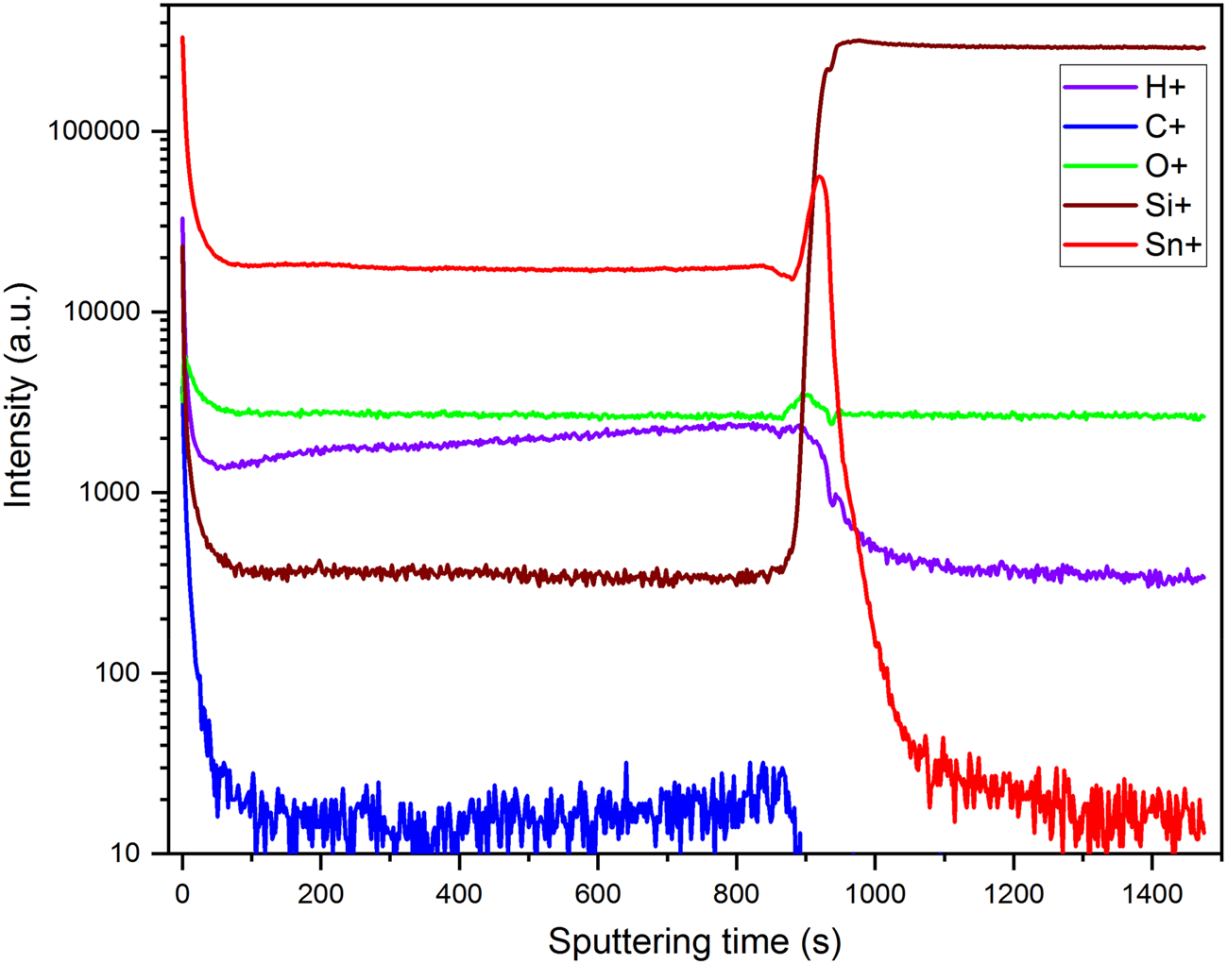
TOF-SIMS profiles for the best conductive sample (SnO_x_ deposited at 250 °C, at 1.5% O_2_/Ar ratio, and annealed under vacuum at 400 °C).

After having investigated the structural properties with XRD and TEM, we further confirm the homogeneity of the SnO_x_ thin film by performing TOF-SIMS on the best conductive sample to show the presence of high quality SnO_x_ by the two constant intensities of Sn and O in the ToF-SIMS steady state conditions between the surface and the interface as shown in Fig. 6. This analysis reveals the constant stoichiometry throughout the depth. The ion yield is much higher for Sn at the surface and the interface due to the matrix effect where the chemical environment changes as the secondary ion yields are strongly dependent on the chemical environment, which explains the high intensity of Sn at the surface and the interface. Si intensity is also much higher at the surface due to a combination of some surface contamination and the higher ion yield at the surface as described previously. SIMS in general is inherently not a quantitative measurement technique. The secondary ion yields are strongly dependent on the chemical environment (matrix effect) and therefore, there is no direct correlation of elemental/compound intensity vs concentration. This technique also revealed the presence of H which slightly increases from the surface to the interface^76^. TOF-SIMS also confirmed the absence of organic or inorganic contamination throughout the depth and it shows also perfect interfaces between the SnO_x_ thin film and the quartz substrate.

In summary, for as deposited SnO_x_ and based on the characterization and discussion, it is suggested that SnO_x_ is predominantly amorphous and/or showing low crystalline SnO in absence of O_2_, owing to the nature of the sputtering target (i.e. SnO). This has led to a lower electrical conductivity. At low O_2_/Ar ratios, the predominating phase become SnO_2_ and the crystallinity tended to improve at higher temperature of 250 °C as the O_2_/Ar ratio increased from 0.5 to 1.5%. The poor oxygen condition is suggested to form defects within SnO_x_ thin film particularly oxygen vacancies and these defects are expected to decrease while increasing the O_2_/Ar ratio. This has led to a higher electrical conductivity. At higher O_2_/Ar ratios, the crystallinity decreased at higher temperature of 250 °C. The rich O_2_ condition is expected to reduce the oxygen vacancy defects and eventually decreases the charge carrier concentration. This has led to a lower electrical conductivity. Both annealing processes are expected to improve the crystallinity of the films due to the thermal treatment at 400 °C for 1 h. However, vacuum annealing is expected to conserve the charge carriers concentrations by preventing annihilation of the oxygen vacancies due to the lack of O_2_. On the other hand, air annealing is expected to reduce the charge carriers concentrations by filling the oxygen vacancies with oxygen supplied from air.

The air annealing has shown a better crystallinity compared to vacuum annealing as all the samples have reported high crystallinity and two SnO_x_ samples deposited without O_2_ recorded the highest crystallite size. Both SnO_x_ deposited without O_2_ and annealed in air have revealed that the presence of both phases SnO and SnO_2_, and it is clear that O_2_ from the air atmosphere has oxidized significantly SnO to SnO_2_. Moreover, as per crystallite size Table S2, we found that the reported crystallite size is relatively higher after vacuum annealing the SnO_x_ samples (deposited at 250 °C). While for the annealing under vacuum (i.e. absence of O_2_), the charge carrier concentration which is mainly due to O vacancies is expected to be conserved. The combination of improved crystallite size and the conservation of charge carrier are the key factors for improving the electrical conductivity as compared to SnO_x_ samples from other series with same O_2_/Ar ratio.

## Conclusions

Our study highlighted the structure/performance correlations of SnO_x_ thin films grown by RF MS. High quality SnO_x_ samples were prepared using magnetron sputtering deposition method followed by thermal annealing processes. Crystalline microstructure, electrical and optical properties were characterised in-depth. Both SnO_2_ and mixed SnO/SnO_2_ thin films were synthetized using RF sputtering. The most electrically conductive sample was obtained by using O_2_/Ar = 1.5% during the growth at 250 °C followed by a moderate vacuum post annealing at 400 °C/5 × 10^–4^ Torr, and has shown a compact and dense morphology without presence of pinholes or cracks, and its grain size were relatively larger compared to other samples, which clearly improved the electron mobility. Its average optical transmittance between 400 and 700 nm was measured to be above 80%. The best optical transmittance of 91% is achieved only using the highest O_2_/Ar ratio of 7.5% for deposited SnO_x_ at 250 °C without annealing and with vacuum annealing as well as both air annealed samples. Vacuum annealing provided a higher electrical conductivity compared to the as-deposited and air-annealed processes. This is attributed to the improvement of crystalline microstructure as well as the presence of oxygen lattice vacancies which has led to a high charge carrier concentration. These growth conditions summarise a good compromise between a high grain size, higher crystalline structure, and high charge carrier concentration.

## Supplementary Information

Supplementary Information.

## Data Availability

The data are available from the corresponding author upon a reasonable request.
