# Supplementary material for: Study of wide bandgap SnOx thin films grown by a reactive magnetron sputtering via a two-step method
Source: Sci Rep. 2022 Sep 12;12:15294. doi: 10.1038/s41598-022-19270-w (PMC9468343; doi:10.1038/s41598-022-19270-w)
Supplement: Supplementary file 1 — Supplementary Information. [file 41598_2022_19270_MOESM1_ESM.docx]

**Supplementary Material**

**Study of Wide Bandgap SnO_x_ Thin Films Grown by a Reactive Magnetron Sputtering via a Two-Step Method**

Y. Zakaria^1,2^, B. Aïssa^1*^, T. Fix^2^, S. Ahzi^2^, A. Samara^1^, S. Mansour^1^, A. Slaoui^2^

*^1^Qatar Environment and Energy Research Institute (QEERI), Hamad Bin Khalifa University (HBKU), Qatar Foundation, P.O. Box 34110, Doha, Qatar*

*^2^Laboratoire ICube - CNRS, Université de Strasbourg, 67000 Strasbourg, France*

**Corresponding authors E-mail: baissa@hbku.edu.qa*

**Diagram S1:** Samples preparation, growth and annealing conditions

| Sample | Substrate Temp (°C) | O2/Ar% (Ar:O_2_ sccm) | Depos. Pressure (10^-3^ Torr) | Thickness (nm) |
| --- | --- | --- | --- | --- |
| S0 | 100 | 0 (200:0) | 4.4 | 100 |
| S1 |  | 0.5 (200:1) | 4.5 | 242 |
| S2 |  | 1.5 (200:3) | 4.6 | 255 |
| S3 |  | 2.5 (200:5) | 4.7 | 277 |
| S4 |  | 4.5 (200:9) | 5 | 199 |
| S5 |  | 7.5 (200:15) | 5.1 | 153 |
| E0 | 250 | 0 (200:0) | 4.4 | 143 |
| E1 |  | 0.5 (200:1) | 4.5 | 170 |
| E2 |  | 1.5 (200:3) | 4.6 | 149 |
| E3 |  | 2.5 (200:5) | 4.7 | 181 |
| E4 |  | 4.5 (200:9) | 4.9 | 108 |
| E5 |  | 7.5 (200:15) | 5.1 | 163 |

**Table S1:** Summary of the RF sputtering conditions and thickness of samples.

| **O_2_/Ar ratio (%)** | **Crystallite Size (Å)** | | | | | |
| --- | --- | --- | --- | --- | --- | --- |
|  | **Deposition Temperature (**°**C)** | | | | | |
|  | **100** | **250** | **100** | **250** | **100** | **250** |
|  | As deposited | | Vacuum Annealing at 400 °C | | Air Annealing at 400 °C | |
| **0** | * | * | * | * | 116 | 109 |
| **0.5** | * | * | * | * | 51 | 54 |
| **1.5** | * | 56 | * | 61 | 56 | 52 |
| **2.5** | * | 54 | * | 56 | 62 | 53 |
| **4.5** | * | 45 | * | 56 | 61 | 55 |
| **7.5** | * | * | * | 56 | 57 | 55 |

**Table S2:** Crystallite size for SnO_x_ sample using Scherrer equation for peak (110).


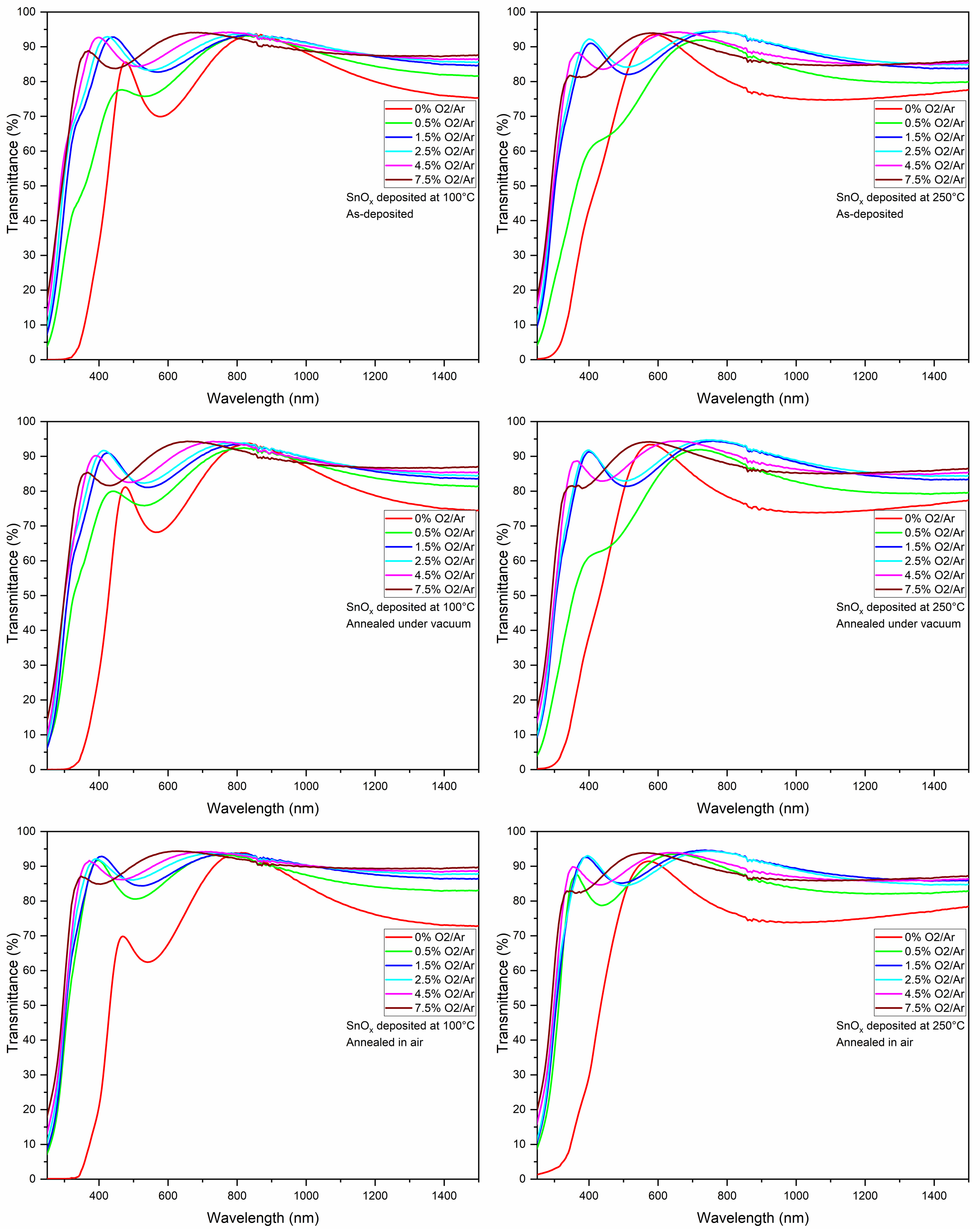


**Figure S1:** UV-Visible transmittance as a function of the wavelength for all the measured SnO_x_ samples.


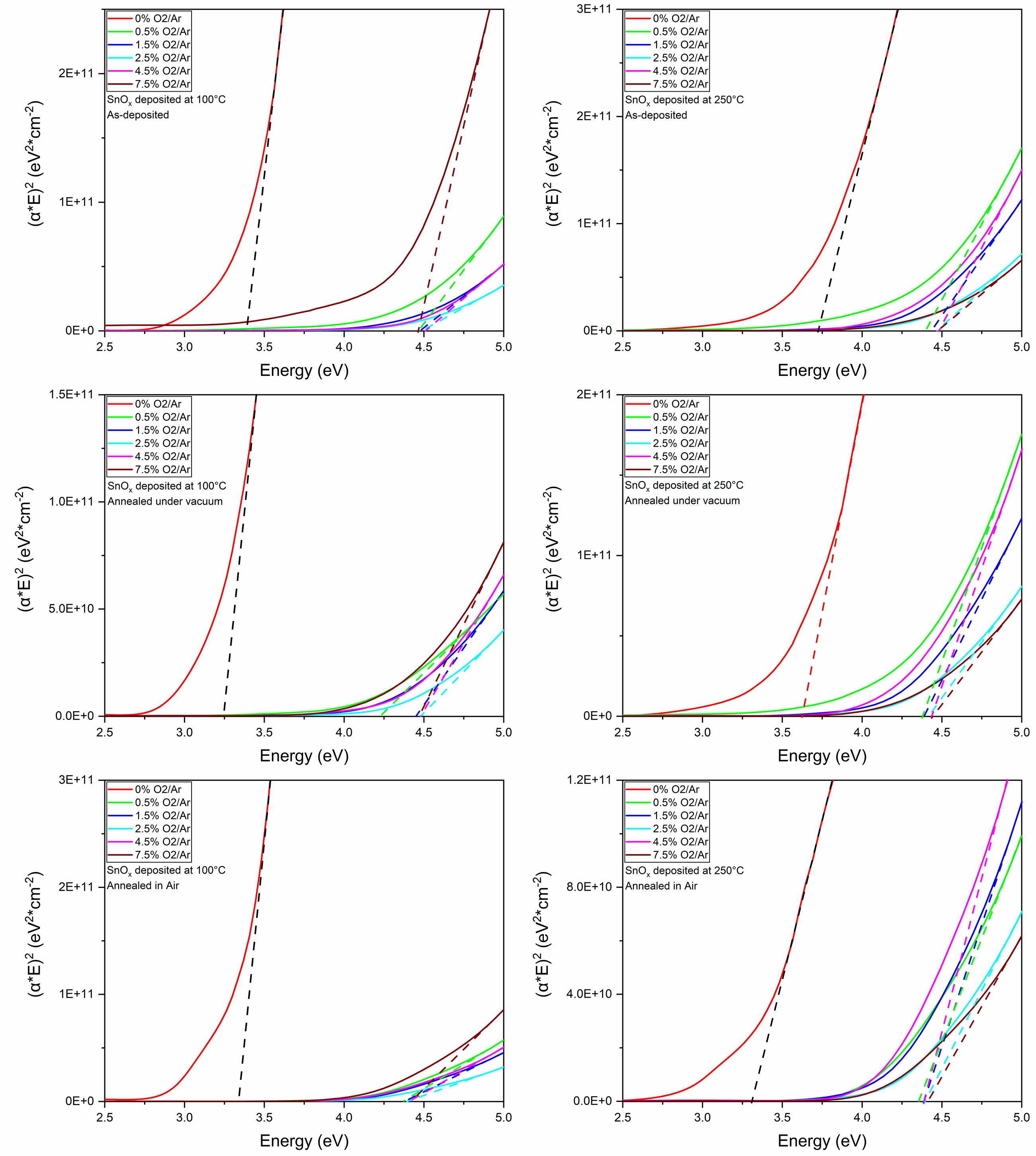


**Figure S2:** Tauc plots for all the measured SnO_x_ samples showing the respective optical bandgaps.

| **FoM Ref.** | **Material** | **Synthesis method** | **Doping** | **Eg (eV)** | **resistivity (Ohm.cm)** | **Sheet resistivity (Ohm/)** | **T%** | **FoM (10^-3^ Ohm^-1^)** |
| --- | --- | --- | --- | --- | --- | --- | --- | --- |
| 1^(1)^ | SnO_2_:Sb | Spray Pyrolysis | Yes | 3.82 | 5.8 x 10^-5^ | 0.61 | 88 | 47.2 |
| 2^(2)^ | SnO_2_:Ba | Spray Pyrolysis | Yes | 3.85 | 6.95 × 10^-4^ | 69 (calculated) | 90 | 5.02 |
| 3^(3)^ | SnO_2_:Sb | Spray Pyrolysis | Yes | 3.77 | 1.16 x 10^-3^ | 141 (calculated) | 85 | 1.39 |
| 4^(4)^ | SnO_2_:Sb | Spray Pyrolysis | Yes | 3.81 | 7.89 x 10^-4^ | 24.9 | 89 | 12.5 |
| 5^(5)^ | SnO_2_:Ta | Spray Pyrolysis | Yes | 3.77 | 4.55 x 10^-4^ | 25.1 | 81 | 6.15 |
| 6^(6)^ | SnO_2_ | Spin Coating | No | 3.92 | 2.63 x 10^-3^ | 63 | 85 | 3.3 |
| 7^(7)^ | SnO_2_:Ti | Sol Gel Spin Coating | Yes | 3.83 | 1.77 x 10^-3^ | 42.3 | 83 | 3.66 |
| 8^(8)^ | SnO_2_:F | Spray Pyrolysis | Yes | NA | 8 x 10^-4^ | 40 | 89 | 2 |
| 9^(9)^ | SnO_2_:F | Sol Gel Dip Coating | Yes | 3.91 | 7 x 10^-4^ | 14.5 | 90 | 24.3 |
| 10^(10)^ | SnO_2_:F | Chemical Vapour Deposition / Pulsed Laser Annealed | Yes | NA | NA | 8.8 | 85 | 23.2 |
| 11^(11)^ | SnO_2_:Te | Pulsed Laser Deposition | Yes | 3.5 | 0.2 | 2.22 x 10^4^ | 87 | 0.012 |
| 12^(12)^ | SnO_2_:La | Spray Pyrolysis | Yes | 3.86 | NA | 2.1 | 80 | 20 |
| 13^(13)^ | SnO_2_:Li | Spray Pyrolysis | Yes | 3.89 | 1.19 | 2.05 | 80 | 0.02 |
| 14^(14)^ | SnO_2_:F | Atmospheric Pressure Chemical Vapour Deposition | Yes | 4.15 | 8.4 x 10^-4^ | 10.5 | 84 | 16.7 |
| 15^(15)^ | SnO_2_:F | Spray Pyrolysis | Yes | NA | 4 x 10^-4^ | 3.71 | 87 | 61.8 |
| 16^(16)^ | SnO_2_:Ta | Spray Pyrolysis | Yes | 4.01 | 4.36 x 10^-4^ | 17.96 | 85 | 47.3 |
| 17^(17)^ | SnO_2_:F | Spray Pyrolysis | Yes | NA | 1.14 x 10^-3^ (calculated) | 7.48 | 65 | 1.93 |
| 18^(18)^ | SnO_2_:GO | Spray Pyrolysis | Yes | 3.69 | NA | 26 | 77 | 2.83 |
| 19^(19)^ | SnO_2_:Gd | Spray Pyrolysis | Yes | 3.63 | 1.02 x 10^-3^ | 27 | 86 | 8.2 |
| 20^(20)^ | SnO_2_ | Sputtering | No | 3.86 | 4.45 x 10^-3^ | 287 (calculated) | 84 | 0.57 |
| 21^(21)^ | SnO_2_:Sb | Spray Pyrolysis | Yes | NA | 1.22 x 10^-3^ | 15.42 | 71 | 2.11 |
| 22^(22)^ | SnO_2_:As | Chemical Vapour Deposition | Yes | 3.87 | 1.5 x 10^-4^ | 15 | 85 | 13.1 |
| 23^(23)^ | SnO_2_:Sb | Spray Pyrolysis | Yes | 3.88 | 8 x 10^-4^ (calculated) | 40 | 88 | 6.9 |
| 24^(23)^ | SnO_2_:F | Spray Pyrolysis | Yes | 4.07 | 3.8 x 10^-4^ (calculated) | 20 | 93 | 24.19 |
| Present Work | SnO_2_ | Sputtering | No | 4.4 | 7.2 x 10^-2^ | 4.8 x 10^4^ | 87 | 0.05 |

**Table S3:** Summary of the FoM values and SnO_x_ electrical and optical properties selected from relevant literature ^(1-23)^.

**Reference:**

1. Fauzia V, Yusnidar MN, Lalasari LH, Subhan A, Umar AA. High figure of merit transparent conducting Sb-doped SnO2 thin films prepared via ultrasonic spray pyrolysis. Journal of Alloys and Compounds. 2017;720:79-85.

2. Islam MA, Mou JR, Hossain MF, Hossain MS. Highly transparent conducting and enhanced near-band edge emission of SnO2:Ba thin films and its structural, linear and nonlinear optical properties. Optical Materials. 2020;106:109996.

3. Hossain MF, Shah MAH, Islam MA, Hossain MS. Transparent conducting SnO2 thin films synthesized by nebulized spray pyrolysis technique: Impact of Sb doping on the different physical properties. Materials Science in Semiconductor Processing. 2021;121:105346.

4. Ramarajan R, Kovendhan M, Thangaraju K, Paul Joseph D. Substrate Temperature Dependent Physical Properties of Spray Deposited Antimony-Doped SnO2 Thin Films. Thin Solid Films. 2020;704:137988.

5. Ramarajan R, Purushothamreddy N, Dileep RK, Kovendhan M, Veerappan G, Thangaraju K, et al. Large-area spray deposited Ta-doped SnO2 thin film electrode for DSSC application. Solar Energy. 2020;211:547-59.

6. Sivakumar P, Akkera HS, Ranjeth Kumar Reddy T, Srinivas Reddy G, Kambhala N, Nanda Kumar Reddy N. Influence of Ga doping on structural, optical and electrical properties of transparent conducting SnO2 thin films. Optik. 2021;226:165859.

7. Sivakumar P, Akkera HS, Kumar Reddy TR, Bitla Y, Ganesh V, Kumar PM, et al. Effect of Ti doping on structural, optical and electrical properties of SnO2 transparent conducting thin films deposited by sol-gel spin coating. Optical Materials. 2021;113:110845.

8. Tarighi A, Mashreghi A. Dependence of Photovoltaic Properties of Spray-Pyrolyzed F-Doped SnO2 Thin Film on Spray Solution Preparation Method. Journal of Electronic Materials. 2019;48(12):7827-35.

9. Tran Q-P, Fang J-S, Chin T-S. Properties of fluorine-doped SnO2 thin films by a green sol–gel method. Materials Science in Semiconductor Processing. 2015;40:664-9.

10. Li B-j, Wang Y-y, Huang L-j, Cao H-d, Wang Q, Ding H, et al. Influences of ultrasonic vibration on morphology and photoelectric properties of F-doped SnO2 thin films during laser annealing. Applied Surface Science. 2018;458:940-8.

11. Chan y Díaz E, Camacho JM, Duarte-Moller A, Castro-Rodríguez R, Bartolo-Pérez P. Influence of the oxygen pressure on the physical properties of the pulsed-laser deposited Te doped SnO2 thin films. Journal of Alloys and Compounds. 2010;508(2):342-7.

12. Mrabet C, Boukhachem A, Amlouk M, Manoubi T. Improvement of the optoelectronic properties of tin oxide transparent conductive thin films through lanthanum doping. Journal of Alloys and Compounds. 2016;666:392-405.

13. Joseph DP, Renugambal P, Saravanan M, Raja SP, Venkateswaran C. Effect of Li doping on the structural, optical and electrical properties of spray deposited SnO2 thin films. Thin Solid Films. 2009;517(21):6129-36.

14. Talaty NN, Beck K, Citeau H, Kirschbaum K, Giolando DM. Characterization of Tin(IV) Oxide Thin Films Prepared by Atmospheric Pressure Chemical Vapor Deposition of cis-[SnCl4{OC(H)OC2H5}2]. Zeitschrift für anorganische und allgemeine Chemie. 2009;635(1):53-63.

15. Moholkar AV, Pawar SM, Rajpure KY, Bhosale CH. Effect of concentration of SnCl4 on sprayed fluorine doped tin oxide thin films. Journal of Alloys and Compounds. 2008;455(1):440-6.

16. Ramarajan R, Fernandes JM, Kovendhan M, Dasi G, Reddy NP, Thangaraju K, et al. Boltzmann conductivity approach for charge transport in spray-deposited transparent Ta-doped SnO2 thin films. Journal of Alloys and Compounds. 2022;897:163159.

17. Moholkar AV, Pawar SM, Rajpure KY, Patil PS, Bhosale CH. Properties of highly oriented spray-deposited fluorine-doped tin oxide thin films on glass substrates of different thickness. Journal of Physics and Chemistry of Solids. 2007;68(10):1981-8.

18. El Radaf IM, Abdelhameed RM. Surprising performance of graphene oxide/tin dioxide composite thin films. Journal of Alloys and Compounds. 2018;765:1174-83.

19. Adjimi A, Aida MS, Attaf N, Ocak YS. Gadolinium doping effect on SnO2 thin films optical and electrical properties. Materials Research Express. 2019;6(9):096405.

20. Belayachi W, Ferblantier G, Fix T, Schmerber G, Rehspringer J-L, Heiser T, et al. SnO2 Films Elaborated by Radio Frequency Magnetron Sputtering as Potential Transparent Conducting Oxides Alternative for Organic Solar Cells. ACS Applied Energy Materials. 2022;5(1):170-7.

21. Babar AR, Shinde SS, Moholkar AV, Bhosale CH, Kim JH, Rajpure KY. Physical properties of sprayed antimony doped tin oxide thin films: The role of thickness. Journal of Semiconductors. 2011;32(5):053001.

22. Vishwakarma SR, Upadhyay JP, Prasad HC. Physical properties of arsenic-doped tin oxide thin films. Thin Solid Films. 1989;176(1):99-110.

23. Singh SK, Basu S. Characterisation of conducting SnO2layers deposited by modified spray pyrolysis technique. Materials Chemistry and Physics. 1988;20(4):381-96.
